# Synthesis of a Novel and Salt Sensitive Superabsorbent Hydrogel Using Soybean Dregs by UV-Irradiation

**DOI:** 10.3390/ma11112198

**Published:** 2018-11-06

**Authors:** Yisa Fan, Mingyue Zhang, Linjian Shangguan

**Affiliations:** 1 School of Mechanical Engineering, North China University of Water Resources and Electric Power, Zhengzhou 450000, China; shangguanlinjian@ncwu.edu.cn; 2 College of Tobacco Science, Henan Agricultural University, Zhengzhou 450002, China; mingyuezhang@henau.edu.cn

**Keywords:** absorption, salt sensitive, soybean dregs, UV-irradiation, kinetics, dwelling

## Abstract

A biomass based hydrogel soybean dregs-Poly(acrylic acid) (SD-PAA) was synthesized under UV radiation while using agricultural waste soybean dregs. Maximum absorption of SD-PAA is 3587 g·g^−1^ in distilled water and 302.0 g·g^−1^ in 150 mM NaCl aqueous solution. Moreover, the influence of granularity, salt solution, and ions in the solutions on water absorption is systematically studied. Sensitivity sequence of the hydrogel to cations was K^+^ < Na^+^ < NH_4_^+^ < Al^3+^ < Fe^3+^ < Mg^2+^ < Ca^2+^, and that to anions was PO_4_^3−^ > SO_4_^2−^ > Cl^−^. Moreover, the experimental results showed that SD-PAA water retention capability remained 37% after centrifugating for 60 min and 0.2% being dried at 60 °C for 70 h. Meanwhile, the swelling data agree well with the pseudo-second-order kinetic model and Fickian diffusion mechanism.

## 1. Introduction

With a three-dimensional cross-linked structure, superabsorbent polymers (SAP) can absorb and store water or liquid that 10 times to thousand times of their own weight. Because of their excellent performance, SAP were widely used in many specialized fields, e.g., farming and gardening [1,2,3], hygienic products [4], medicine [5,6], industrial dewatering, architecture [7,8], sewage disposal [9,10], and metal ion removing [11,12]. SAP’s worldwide market stood at 1861.8 kilo tons in 2013, valued at 6.06 billion United States (US) dollars, and will reach 8.78 billion US dollars by 2020 [13]. Demands of the market make researchers pay more and more attention to them.

The monomers types of synthesis of SAP are mainly acrylic acid, acrylonitrile, acrylamides, and polyvinyl alcohol, etc. Among these raw materials, acrylic acid controls most of the market share because of its low cost, safe, reliable, and simple synthesis process. Nevertheless, with environmental concerns and volatility in prices of acrylic acid, considerable interest has been inspired in bio-based natural materials superabsorbent. Moreover, bio-based superabsorbent also has the advantages of excellent water absorbent capacity, good biocompatibility, and biodegradability [14]. Bio-based natural materials, such as wheat straw, maize, and cassawa starch, were used to prepare SAP [15,16,17]. There are few literatures in SAP synthesis using soybean dregs. As an important food crop, soybean has a huge annual production. According to US Dept. of Agriculture, in 2015–2016 year, the global soybean output will reach 317.3 million tons [18]. Soybean dregs (SD) are by-product of soybean, with large production but low utilization. Today’s resource and energy problem have become increasingly prominent, so the research on effective use of available resources is particularly meaningful. Above all, abundant production, low prices, and high active ingredients make SD an ideal stuff for synthetic SAP.

Nowadays, methods of solvent polymerization [19,20] and suspension polymerization [21,22] are generally used to prepare SAP. However, there are some shortcomings, such as long synthetic process, inconvenient operation, and requiring exorbitant specialized equipment, which may increase the cost of production. In order to solve those problems, some new methods come out in recent years, for instance, microwave irradiation [23], UV irradiation, 15 γ irradiation [24], and glow discharge electrolysis plasma irradiation [25]. During these methods, as a simple, convenient, and efficient way, UV irradiation causes much attention to the researchers.

In this study, we find a new method to utilize SD. Using these SD, a new kind of SPA is prepared under UV irradiation for the first time. Because of the low cost of raw-material and simple, fast synthetic process, the production cost had been reduced. More importantly, there are rarely researches on the effects of various ions on water absorption especially that on bio-based polymers. The structure, morphology and thermal stability of soybean dregs-Poly(acrylic acid) (SD-PAA) hydrogels have been discussed. Meanwhile, a reaction mechanism of SD-PAA hydrogels is suggested also. The effects of solution concentration, ionic strength and different ions on water absorption have been studied systematically. What is more, water retention capacity and effect of granularity on water absorption is investigated. Moreover, swelling kinetics and diffusion kinetics in distilled water and 150mM NaCl solution are detailedly studied.

## 2. Materials and Methods

### 2.1. Chemicals and Reagents

Soybean dregs (SD, Jilin Agricultural University, Changchun, China) was dried and crushed then sieved through a 120 mesh steel screen before use. SD we used contains 26.1% of crude cellulose, 12.8% of crude protein, 9.38% of moisture, 2.05% of nitrogen element, and 2.77% of crude fat. Acrylic acid (AA) and Ammonium persulfate (APS) were purchased from Fuchen Chemical Reagents (Tianjin, China). *N*,*N*′-methylenebis(acrylamide) (MBA), benzoin dimethyl ether (BDK), methyl alcohol, NaOH, and NaCl were supplied *N*,*N*′-methylenebis(acrylamide) by Beijing Chemical Works (Beijing, China). All of the chemicals were analytical grade.

### 2.2. SD-PAA and Poly(acrylic acid) (PAA) Preparation

SD, APS, BDK, and MBA (m(AA):m(SD):m(MBA):m(APS):m(BDK) = 100:60:0.2:0.10:1.25) were mixed in 2.0 mL 80% neutralization degrees of AA solution (It was obtained by neutralization reaction of AA and 20 wt. % NaOH solution. It is equal to 0.6 g of AA). The mixtures were processed using ultrasonic for about one minute. Then, put the homogenous mixtures under a 1000 W UV lamp (Shenzhen Bofeida science and technology, Shenzhen, China) (wavelength of 365 nm) for 10 min, maintaining the distance for 37 cm between the lamp and reaction mixture. After that, the product was soaked in methyl alcohol for 12 h, and then filtered it and dried (70 °C) to a constant weight. Finally, sieve the dry product through 20, 60, and 80 mesh steel screen and set them aside for use.

PAA was synthesized according to the above method, except that no SD.

### 2.3. Characterization

Elemental analysis of the samples was evaluated using a Vario EL cube Elementar (Elementar Analysensysteme GmbH, Hanau, Germany). SEM were recorded on a SSX–550 SEM instrument (SHIMDZU, Kyoto, Japan) after coating the samples with gold using ETD–2000 auto sputter coater (ETDC, Ltd., Avion, France). FTIR spectra were obtained from a 1.50SU1 spectrometer (SHIMDZU, Kyoto, Japan) using KBr pellets. Thermogravimetric Analysis (TGA) were obtained from a PerkinElmer Pyris 1 TGA thermogravimetric analyzer (PerkinElmer, Waltham, MA, USA), in the temperature range of 25–850 °C at a heating rate of 10 °C min^−1^ under a flowing nitrogen atmosphere.

### 2.4. Water Absorption Capacity

Gravimetric analysis was used to determine water absorption of soybean dregs-Poly(acrylic acid) (SD-PAA). Firstly, immerse 0.10 g dry SD-PAA in 500 mL solution (distilled water or salt solutions) and magnetic stirring for *t* min at 25 °C. Secondly, the swollen gel was filtered through a 100-mesh nylon bag, and the bag was suspended for 30 min until dripping particularly slow or almost no water drops down. Then, the swollen gels were weighed and the water absorption was calculated using Equation (1):

(1) $$Q_{t}=\frac{m_{t}-m_{0}}{m_{0}}$$

where *Q_t_* (g·g^−1^) was the water absorption at time *t* (min) and *m*_0_ (g) was the weight of dry SD-PAA. *m_t_* (g) was the weight of swollen gel at time *t* (min). *Q_e_* was the water absorption at equilibrium state.

Water Retention Capacity. Approximately 40 g swollen gels (*M*_0_, g) were centrifuged under 6000 rpm (centrifugal radius = 8.6 cm) or dried under 60 °C for *t* min. The water retention capacity (*WR*) was calculated by Equation (2):

(2) $$WR\left(\%\right)=\frac{M_{t}}{M_{0}}\times 100$$

where *M_t_* (g) was the weight of remaining gels after centrifuged or dried for *t* mins.

## 3. Results and Discussion

### 3.1. Mechanism of SD-PAA

**Figure 1:**
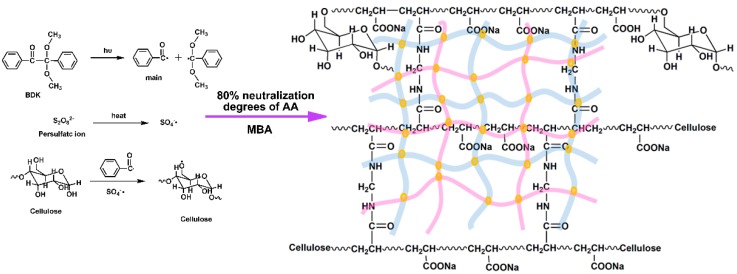
Schematic illustrations of the preparation of soybean dregs-Poly(acrylic acid) (SD-PAA).

Synthesis Mechanism of SD-PAA. The mechanism for preparation of SD-PAA was showed in Figure 1. Mainly included three steps: 1. Chain initiation, under the UV lamp, the initiators decomposed into primary radicals of benzoyl and sulfate radicals. These primary radicals swap out H from –OH of cellulose in SD to form alkoxy radicals. 2. Chain propagation, alkoxy radicals reacted with AA molecules to form new radicals. These new free radicals reacted with other monomers, which caused the chain growth. During the process of chain growth, the vinyl groups of MBA reacted with the chains, forming a cross-linked network structure. 3. Chain termination, when the polymer monomers were exhausted, the interaction between free radicals made the chain reaction end.

### 3.2. Characterization

#### 3.2.1. Elemental Analysis

Elemental analysis (Table 1) was employed to determine the contribution of SD to SD-PAA. When comparing with PAA, the content of H, C, and N in SD-PAA increased from 4.97, 33.84, and 0.13% to 5.00, 37.11, and 0.53%, respectively. This was due to that the cellulose molecules and N containing compound (protein) in SD reacted with AA monomer, which led to the increase of C and N contents. This also proved that SD participated in the reaction to form the polymer chain.

#### 3.2.2. SEM Analysis

**Figure 2:**
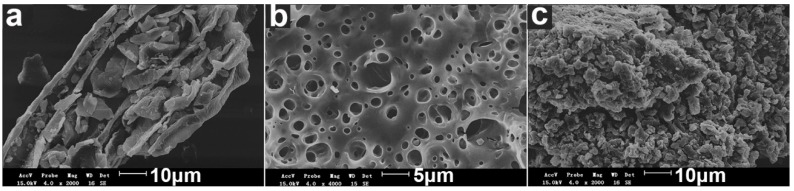
SEM images of the surfaces of (**a**) SD; (**b**) PAA, and (**c**) SD-PAA.

The morphology of SD, PAA, and SD-PAA were investigated according to SEM analysis (Figure 2a–c). As shown in Figure 2a, SD presented a loose and thin layer shape. Figure 2b showed that the surface of PAA was tight and smooth, being distributed irregular circular holes. However, SD-PAA presented a totally different morphology with PAA. Firstly, it was comparatively loose and coarse, which increased the specific surface area of the material greatly. Secondly, a lot of irregular gaps structures distributed in both internal and external material. Based on the above two reasons, it was more advantageous to facilitate water permeation into the polymeric network. It also suggests that the cellulose molecules in SD had reacted with the polymer chains.

#### 3.2.3. FITR Analysis

**Figure 3:**
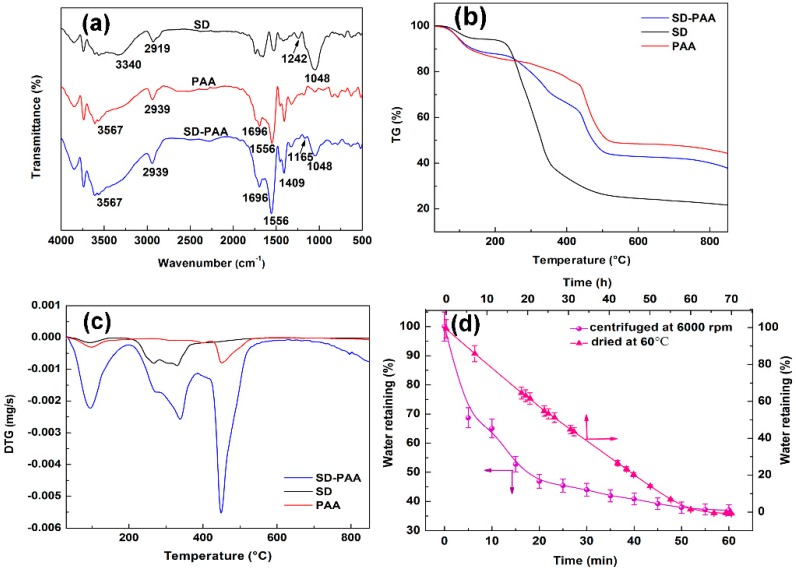
(**a**) FTIR spectra of SD, PAA and SD-PAA; (**b**) Thermogravimetric (TG) curves of SD, PAA, and SD-PAA; (**c**) DerivativeThermogravimetry (DTG) curves of SD, PAA, and SD-PAA, and (**d**) Water retaining of SD-PAA.

The FTIR spectra of the samples are shown in Figure 3a. For SD, the characteristic peaks assignment of cellulose were 3340 cm^−1^ (stretching vibrations of O–H), 2919 cm^−1^ (C–H stretching vibrations), 1242 cm^−1^, and 1048 cm^−1^ (–C–O–C and –C–O stretching vibrations of polysaccharide ring) [26,27]. In the case PAA and SD-PAA, the peak at 3567 cm^−1^ was related to O–H and N−H bonds, peaks at 2939, 1696, and 1409 cm^−1^ were owing to C–H, C−N stretching vibration, and –COO^−^ symmetric stretching, respectively [28]. The peak at 1556 cm^−1^ was related to C=O and amide II band. In the case SD-PAA, additional peaks at 1165 cm^−1^ and 1048 cm^−1^ was observed than PAA, which belong to –C–O–C (compared with SD, the peak shifted to a smaller value) and –C–O. The appearance of the two peaks illustrates the reaction of SD and the polymer chains, confirming the successful grafting of SD to PAA chains.

#### 3.2.4. TGA Analysis

The TG/DTG analysis of the samples was shown in Figure 3b,c. The TG curve of SD show that the pyrolysis process for SD consisted of two main steps: 25–214 and 214–548 °C. The weight loss of the first stage was 6.26, which due to the evaporation of moisture adsorbed from the atmosphere and the escape of a small amount of hydroxyl. The mass loss in the second stage was 68.45%, which was mainly because the decomposition of protein, cellulose, and hemicellulose [29]. In this stage, glycosidic bond began to fracture, cellulose and hemicellulose molecules depolymerized. As the DTG curve of SD showed, their corresponding maximum decomposition rate appeared at 177 and 353 °C. The TG curve of PAA exhibited four steps: 25–176, 176–423, 423–535, and 535–850 °C, with weight loss of 11.71, 12.66, 26.49, and 4.52%, respectively. For the TG curve of SD-PAA, the weight loss (approximately 11.60%) of the first stage occurred in 25–194 °C, which due to the moisture evaporation. The second region occurred in 194–384 °C with the weight loss of 20.86%. This weight loss was associated with decomposition of branched chain and breaking of weak chemical bonds, such as hydroxyl, ester, and C–O–C containing in SD-PAA. To be more exact, decomposition of cellulose chain and part of H_2_O and CO_2_ molecule eliminated from the polymeric backbone [27], such as the dehydration of two adjacent carboxylic groups forming anhydride, and the decarboxylation reaction between carboxylate groups [30]. A rapid decomposition rate of SD led to a faster degradation of SD-PAA than PAA in this stage. The major weight loss 24.04% occurring between 384 to 567 °C was the contribution of further oxidation and fracture of crosslinked network. The decomposition of residual organic materials caused 5.69% weightlessness in the last stage 567–850 °C. Ultimately, the remnant weight was 37.81%; this was attributed to residual inorganic salts after polymer decomposition. Due to the addition of SD, the residual amount of SD-PAA was less than PAA (44.62%) and more than SD (22.08%).

### 3.3. Water Retention

Figure 3c was the results of *WR* under the condition of high pressure and high temperature. It displays that the *WR* maintained 37% after centrifugating for 60 min and 0.2% drying at 60 °C for 70 h. *WR* of the superabsorbent polymer affected by hydrogen bond interaction between the composite and H_2_O molecules as well as Van der Waal’s forces [31]. Large amount of carboxylate groups and hydroxyl containing in SD-PAA led to enhanced chemical interaction, thus strengthening *WR*. It can be seen from the curve (centrifuged at 6000 rpm) that in the first 15 min, the water retention declined sharply from 100% to 52.74%, while 15 min later, it became gentlely. The reasons were that, initially, it was easier to break free from the gels for weak-absorption water. Then, as time went on, ratio of weakly absorbed water declined and that of strongly absorbed water increased, which led to a slow water loss. The curve that was dried at 60 °C emerged an analogous property, but more gentle. It indicates that the hydrogels showed a relatively higher sensitivity to pressure than temperature.

### 3.4. Water Absorption

#### 3.4.1. Effect of Cations

This work studied the effects of media solution properties on the water absorption of SD-PAA, found that ion species, ion valence, and ionic strength had a great impact. To achieve the sensitivity of the samples to media solution, a salt sensitivity factor (*f*) was calculated (salt concentration 150 mM) by Equation (3), as follows [32].

(3) $$f=1-\frac{Q_{s}}{Q_{d}}$$

where *Q_s_* was the water absorption in saline and *Q_d_* was that in deionized water.

According to Flory’s equation [33], ionic strength affected the water absorption of polymer greatly.

(4) Q53=(i/2VuI1/2)2+(1/2−χ1)/V1ve/V0

where *Q* was the water absorption of superabsorbent polymers, *v_e_*/*V*_0_ was the crosslinking density, *I* was the ionic strength of saline, *i/V_u_* was the charge density of polymer, and (1/2 − χ_1_)/*V*_1_ was the polymer-solvent affinity.

**Figure 4:**
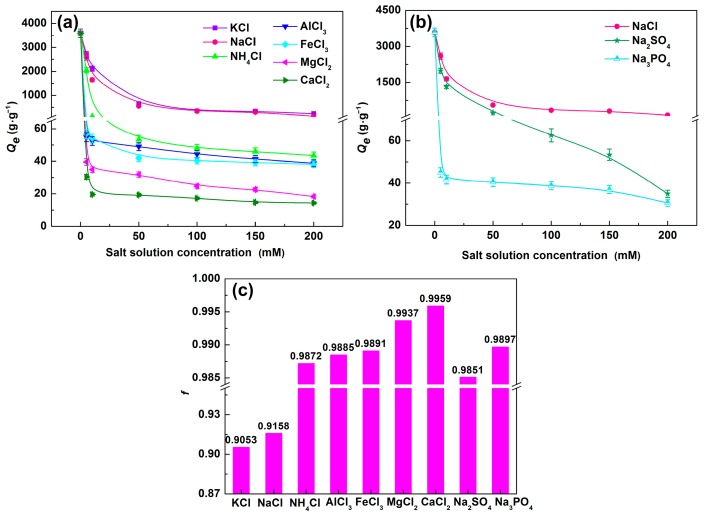
Water absorption of SD-PAA in (**a**) KCl, NaCl, NH_4_Cl, CaCl_2_, MgCl_2_, FeCl_3_, and AlCl_3_; (**b**) NaCl, Na_2_SO_4_, and Na_3_PO_4_ aqueous solutions, and (**c**) salt sensitivity factor (*f*) of different salt with concentration of 150 mM.

The effect of different cations on water absorption of SD-PAA was shown in Figure 4a and Table 2. From Figure 4a, three conclusions can be obtained:

1. The water absorption decreased rapidly in low salt concentration (0–50 mM), and decreased gentlely at the concentration above 50 mM. As an ionic hydrogels, the extra cations in media solution caused increased electrostatic repulsion, and changed the osmotic pressure between the media solution and the polymer, which led to low water absorption [34].
2. The water absorption order in different chloride salts solution was KCl > NaCl > NH_4_Cl > AlCl_3_ > FeCl_3_ > MgCl_2_ > CaCl_2_. The influence of seven cationic on water absorption followed the sequence: K^+^ > Na^+^ > NH_4_^+^ > Al^3+^ > Fe^3+^ > Mg^2+^ > Ca^2+^. The monovalent cationic salts had less effect on water absorption than polyvalent salts. This was due to the fact that divalent and trivalent metal ion can form a complexe with −COO^–^ groups containing in polymer SD-PAA and the polyvalent salt solutions had higher ionic strength; the ionic strength order of 150 mM saline solution was Al^3+^, Fe^3+^ > Mg^2+^, Ca^2+^ > K^+^, Na^+^ (Table 2). According to Equation (4), *Q_e_* decreased with the increase of ionic strength. Interestingly, *Q_e_* in trivalent cationic solution were higher than in divalent cationic solution, while, as shown in Table 2, the ionic strength of trivalent cationic solution was stronger. It may be because that trivalent cationic affected the charge density of polymer more than divalent, which led to stronger water absorption.
3. For monovalent metal ion K^+^ (ionic radius was138 pm) and Na^+^ (ionic radius was102 pm), the larger the radius, the higher water absorption was (K^+^ > Na^+^), while for divalent and trivalent metal ion Ca^2+^, Mg^2+^, Fe^3+^, and Al^3+^ (their ionic radius were 99.0, 72.0, 64.5, and 53.5 pm, respectively), the larger the radius, the less water absorption was (Ca^2+^ < Mg^2+^ < Fe^3+^ < Al^3+^). This may be due to the coordination interaction of these metal ions with the –COO^−^ groups on polymer chains. So, these metal ions acted as crosslinking agents of the polymer, which prompted the polymer, absorbed more water. Moreover, for monovalent cation, the water absorption declined severer in polyatomic solution (NH_4_^+^) than in single-atomic solution. Above all, salt sensitivity of SD-PAA in different cations solution was Ca^2+^ > Mg^2+^ > Fe^3+^ > Al^3+^ > NH_4_^+^ > Na^+^ > K^+^ (Figure 4c).

#### 3.4.2. Effect of Anions

Effects of different valence anions (Cl^−^, SO_4_^2−^ and PO_4_^3−^) with Na^+^ on water absorption were showed in Figure 4b. The water absorption showed a tendency that Cl^−^ > SO_4_^2−^ > PO_4_^3−^. This due to the ionic strength was Na_3_PO_4_ > Na_2_SO_4_ > NaCl at the same molarity. The sensitivity factors of NaCl, Na_2_SO_4_, and Na_3_PO_4_, solution were 0.9158, 0.9851, and 0.9897 at the salt concentration of 150 mM, respectively (Figure 4c). The results indicate that polyvalent anion salt solution showed greater impact on water absorption. Under the same concentration, the gels showed larger sensitivity to multivalent anion than to one-valence anion.

#### 3.4.3. Effect of Particle Size and Salt Solution

**Figure 5:**
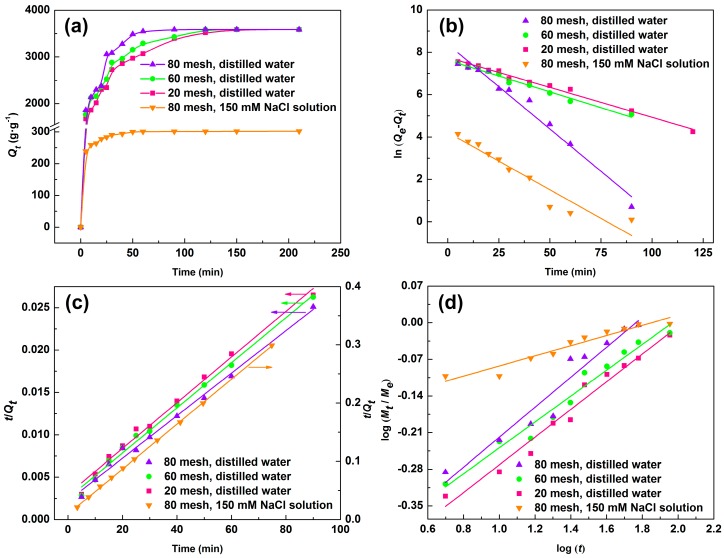
(**a**) Swelling properties of SD-PAA with particle size of 80, 60, and 20 mesh in distilled water and 150 mM NaCl solution; kinetic analysis: (**b**) the pseudo-first-order kinetic model; (**c**) the pseudo-second-order kinetic model and, (**d**) the water diffusion kinetic.

The results of effect of hydrogel particle size and NaCl solution on the water absorption process were shown in Figure 5a. It is clear that the granularity had an obvious effect on water absorption before balance, while almost had no influence on balance absorption. Before the balance, the smaller the particle sizes were, the stronger water absorption, especially in the first 30 min. The reason was that small particles had a relatively large surface area, which determined the contact area with the liquid, resulting high absorbent [35]. The swelling process in distilled water (80 mesh) had three steps: First, 0–25 min, sharp increase stage, which had fastest absorption rate, achieved 85.2% of equilibrium absorption capacity. Second, 25–60 min, slower increase stage, contributions to balance water absorption was about 13.7%. Third, 60–210 min, balance stage, the water absorption almost unchanged. The similar swelling process was applicable to the other granularity (60 mesh and 20 mesh), but lower absorption rate and achieved equilibrium at 120 min. The water absorption process in NaCl solution (150 mM) at particle size of 80 mesh was also examined. Because of the addition of salt, water absorption was reduced greatly, and the equilibrium achieved earlier (50 min).

### 3.5. Kinetic Analysis

#### 3.5.1. Swelling Kinetic

In order to examine the swelling kinetic data of water absorption on SD-PAA, the pseudo-first-order kinetic model and the pseudo-second-order kinetic model were detected to fit the experiment data. The pseudo-first-order kinetic model [36] was represented as Equation (5), which was widely used in solid–liquid absorption.

(5) $$\ln\left(Q_{e}-Q_{t}\right)=\ln Q_{e}-K_{1}$$

The pseudo-second-order kinetic model [37] was expressed as Equation (6), which was based on the equilibrium absorption.

(6) $$\frac{t}{Q_{t}}=\frac{1}{K_{2}{Q_{e}}^{2}}+\frac{t}{Q_{e}}$$

where *Q_e_* (g·g^−1^) was water absorption at equilibrium, *Q_t_* (g·g^−1^) was water absorption for liquid attracting *t* minutes, *K*_1_ (min^−1^) and *K*_2_ (g·g^−1^·min^−1^) were the rate constants. The pseudo-first-order plot, pseudo-second-order plot, and data associated with them were given in Figure 5b,c and Table 3. The correlation coefficient values (*R*^2^) of the pseudo-second-order kinetic were higher than 0.99 for all swelling process and greater than values of the pseudo-first-order kinetic. So, the experimental data fitted better with the pseudo-second-order kinetic. Moreover, the calculated value (*Q_e, cal_*) that was obtained from the pseudo-first-order kinetic model showed quite different with the experimental data (*Q_e, exp_*), and that from the pseudo-second-order kinetic model were close. Thus, swelling behavior of the hydrogel was more suitable for the pseudo-second-order kinetic model.

#### 3.5.2. Diffusion Kinetic

In order to estimate water diffusion behavior of SD-PAA, diffusion kinetic that can be expressed as Equation (7) [38] was used to analyze the experiment data.

(7) log(MtMe)=log(k)+nlog(t)

where *M_e_* and *M_t_* were the mass of water absorption at equilibrium and at liquid attracting *t* minutes. *k* was a characteristic constant of the sample and *n* was a diffusional exponent. The value of *k* and *n* (given in Table 3) were calculated according to the fitting line of log(*M_t_/M_e_*) versus log(*t*) (Figure 5d). The diffusion mechanism was divided into two categories: 1. *n* < 0.5, the outside liquid moving into the network is a major contributor to the swelling of the polymer. It conformed to the Fickian diffusion mechanism; 2. 0.5 < *n* < 1, polymer network relaxation rate ≥ liquid diffusion rate. It conformed to non-Fickian diffusion mechanism. *n* values were less than 0.5 for all samples, which indicated that the liquid diffusion mechanism in line with Fickian diffusion mechanism in distilled water and 150 mM NaCl solution. The liquid that is moving into the network structure was a major cause of the swelling process, which also led to fast absorbing water equilibrium.

## 4. Conclusions

A novel superabsorbent composite (SD-PAA) was prepared by free radical polymerization. SD-PAA showed excellent water absorption performance, and following the pseudo-second-order swelling kinetic model. The particle size, liquid concentration, and anion and cation in different valence had influence on water absorption of SD-PAA. Water absorption declined with the increasing of salt concentration. The mathematical analysis of the water diffusion kinetics shows that the Fickian diffusion was dominant in distilled water and 150 mM salt solution. Moreover, SD-PAA showed excellent water retention performance under high temperature and high pressure. With high water absorption and retention capacity, SD-PAA was expected to be used in agricultural, forestry, and horticulture application.

## Figures and Tables

**Table 1:** Elemental analysis of soybean dregs (SD), Poly(acrylic acid) (PAA), and SD-PAA.

Sample	C (%)	N (%)	H (%)
SD	38.48	0.76	5.17
PAA	33.84	0.13	4.97
SD-PAA	37.11	0.53	5.00

**Table 2:** Effect of saline on the balanced water absorption.

Solution (150 mM)	Ionic Strength ^a^ (mol-ion dm^−3^)	*Q_e_* (g·g^−1^)
KCl	0.15	339.0
NaCl	0.15	302.0
NH_4_Cl	0.15	45.9
CaCl_2_	0.45	14.7
MgCl_2_	0.45	22.6
FeCl_3_	0.90	39.2
AlCl_3_	0.90	41.3
Na_2_SO_4_	0.45	53.4
Na_3_PO_4_	0.90	36.9

^a^$I=0.5\Sigma {\left(C_{i}Z_{i}\right)}^{2}$, where I is the ionic strength, C_i_ is the ionic concentration and Z_i_ is charge on each individual ion.

**Table 3:** Kinetic parameters for swelling behavior of SD-PAA in distilled water and 150 mM NaCl solution.

Sample (mesh)	*Q_e,exp_* g·g^−1^	Pseudo-First Order Kinetic			Pseudo-Second Order Kinetic			Diffusion Kinetic		
		*R* ^2^	*K*_1_ (min^–1^)	*Q_e, cal_* (g g^–1^)	*R* ^2^	*K*_2_ (10^−5^ g·g^−1^·min^−1^)	*Q_e, cal_* (g·g^−1^)	*R* ^2^	*n*	*k*
20 ^a^	3587	0.9899	0.0283	2336	0.9900	2.43	3707	0.9698	0.2643	0.2912
60 ^a^	3587	0.9812	0.0300	2060	0.9933	2.80	3756	0.9668	0.2470	0.3266
80 ^a^	3587	0.9685	0.0800	4343	0.9919	3.84	3984	0.9335	0.2862	0.3127 0.6609
80 ^b^	302.0	0.9036	0.0541	67.6	0.9998	163.3	308.6	0.9260	0.0971	

^a^ The swelling behavior in distilled water; ^b^ the swelling behavior in 150 mM NaCl solution.
